# Rapid Identification of ESKAPE Bacterial Strains Using an Autonomous Microfluidic Device

**DOI:** 10.1371/journal.pone.0041245

**Published:** 2012-07-27

**Authors:** Jack Y. Ho, Nate J. Cira, John A. Crooks, Josue Baeza, Douglas B. Weibel

**Affiliations:** 1 Department of Biomedical Engineering, University of Wisconsin-Madison, Madison, Wisconsin, United States of America; 2 Department of Bioengineering, Stanford University, Stanford, California, United States of America; 3 Department of Biochemistry, University of Wisconsin-Madison, Madison, Wisconsin, United States of America; Texas A&M University, United States of America

## Abstract

This article describes **Bac**teria ID **Chips** (‘BacChips’): an inexpensive, portable, and autonomous microfluidic platform for identifying pathogenic strains of bacteria. BacChips consist of a set of microchambers and channels molded in the elastomeric polymer, poly(dimethylsiloxane) (PDMS). Each microchamber is preloaded with mono-, di-, or trisaccharides and dried. Pressing the layer of PDMS into contact with a glass coverslip forms the device; the footprint of the device in this article is ∼6 cm^2^. After assembly, BacChips are degased under large negative pressure and are stored in vacuum-sealed plastic bags. To use the device, the bag is opened, a sample containing bacteria is introduced at the inlet of the device, and the degased PDMS draws the sample into the central channel and chambers. After the liquid at the inlet is consumed, air is drawn into the BacChip via the inlet and provides a physical barrier that separates the liquid samples in adjacent microchambers. A pH indicator is admixed with the samples prior to their loading, enabling the metabolism of the dissolved saccharides in the microchambers to be visualized. Importantly, BacChips operate without external equipment or instruments. By visually detecting the growth of bacteria using ambient light after ∼4 h, we demonstrate that BacChips with ten microchambers containing different saccharides can reproducibly detect the ESKAPE panel of pathogens, including strains of: *Enterococcus faecalis*, *Enteroccocus faecium*, *Staphylococcus aureus*, *Klebsiella pneumoniae*, *Acinetobacter baumannii*, *Pseudomonas aeruginosa*, *Enterobacter aerogenes*, and *Enterobacter cloacae*. This article describes a BacChip for point-of-care detection of ESKAPE pathogens and a starting point for designing multiplexed assays that identify bacterial strains from clinical samples and simultaneously determine their susceptibility to antibiotics.

## Introduction

The ESKAPE acronym (*Enterococcus faecium*, *Staphylococcus auerus, Klebsiella pneumoniae*, ***A*** *cinetobacter baumannii*, *Pseudomonas aeruginosa*, and the *Enterobacter* species) coined by Rice represents a collection of the most common nosocomial pathogens that escape the effects of many clinical antibiotics [1], [2]. The emergence of the ESKAPE panel, and the growing trend of antimicrobial resistance, has become a serious concern for the Infectious Diseases Society of America (IDSA), who recently issued a “Call to Action for the Medical Community” [1]. Studies show that ESKAPE pathogens are responsible for over 40% of infections in patients in intensive care units [3].

**Figure 1:**
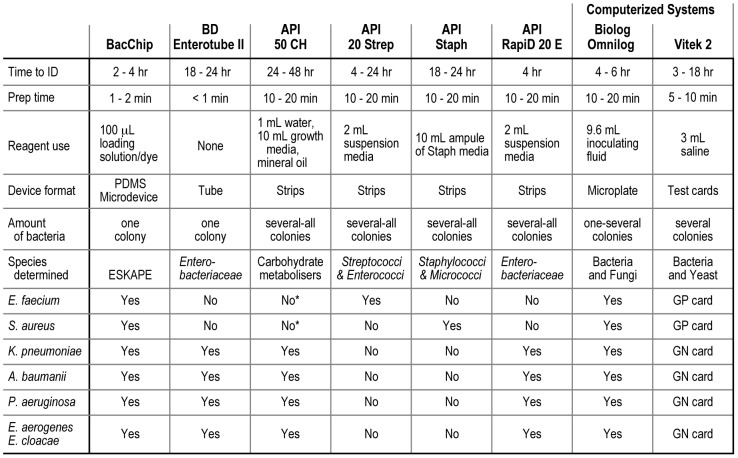
The BacChip versus current commercial diagnostics. A table comparing the BacChip device with commercial diagnostic systems that include comparable self-contained hand-held devices and advanced computerized systems. “Time to ID” refers to the identification time after a bacterium has been isolated and amplified (typically an additional 24–48 h required for all diagnostic devices listed). The designation ‘GN’ and ‘GP card’ for the Vitek 2 indicates that different test cards are required to identify these organisms. *The API 50 CH system is not marketed for Gram-positive bacteria nor does it have a GP database for identification of strains. However, the method of identification of the API 50 CH (carbohydrate metabolism) could in principle be used to determine Gram-positive bacteria.

There are two measurable factors that can guide the treatment of patients with bacterial infections. One factor is the minimum inhibitory concentration (MIC) of an antibiotic against the pathogen. We recently demonstrated a portable, simple-to-use microfluidic device that enables the determination of MIC values for clinical pathogens [4]. The other factor is the identity of the pathogen. Together, these two factors enable pathologists to prescribe antibiotics at doses that minimize the risk of bacteria developing resistance while maximizing therapeutic outcome [5], [6]. In clinical settings, bacterial identification and MIC determinations are performed after the bacterial species has been isolated from a patient sample; obtaining this primary isolate requires an incubation period of 24–48 h [7]. Numerous commercial instruments are available (e.g., DiversiLab DL, Vitek 2, BBL Crystal, Biolog; Figure 1) to identify bacterial species after isolation, and these technologies display a characteristic tradeoff between price and speed. Rapid bacterial identification (<4 h) requires expensive instruments that are costly to operate and maintain [8], [9]. The cost and size of these instruments also restricts their use for point-of-care medicine and necessitates the establishment of centralized labs for high-throughput analysis of clinical samples.

The assays in most modern instruments for bacterial identification are based on well-established phenotypic screens. For example, a commonly used assay tests the fermentation of a panel of saccharides by an unknown bacterium [10]–[12]. A pH indicator or fluorescent dye reveals the fermentation of different saccharides. Comparing this metabolic fingerprint against a library of tabulated fermentation data enables the identification of an organism. Several non-automated, commercial devices use saccharide-based assays (e.g., BD enterotube II and bioMérieux API 50 CH strips). The simplicity of these assays makes them attractive, inexpensive, and robust; they are, however, characteristically slow (requiring an additional ∼18–24 h after the isolation and amplification of bacteria) and may require large sample sizes (e.g., several colonies or an entire lawn of bacteria on a 15 mm diameter Petri dishes may be needed to inoculate the requisite number of wells) [13]. Furthermore, the inoculation of cells into multiple, parallel wells requires multiple pipetting or liquid handling steps that introduce potential sources of error and contamination. In contrast to handheld devices, automated systems such as the Biolog OmniLog and bioMérieux Vitek 2 can identify bacteria rapidly (∼4 h after isolation and amplification of cells from a colony) [14], [15]. These systems, however, are not suitable for distributed or point-of-care diagnostics; they are designed for large, centralized clinical microbiology labs. The introduction of a small, inexpensive, disposable, fast, and technically simple device for identifying bacteria will extend this category of assays to point-of-care settings and increase the throughput of these assays in clinical labs (i.e., one pipetting step and minimal user training).

To develop a portable system for identifying bacteria, we focused our attention on the hands-free technology we developed for measuring the MIC of antibiotics against bacterial strains [4]. In this article we expand the capabilities of this diagnostic system to simplify and accelerate identification assays, and use it to detect the ESKAPE panel of clinical bacteria. Rather than redesigning separate devices for measuring MICs and identifying bacteria, we instead adapt and optimize the same device for both assays. This approach enables us, in principle, to perform both assays simultaneously in the same device.

**Figure 2:**
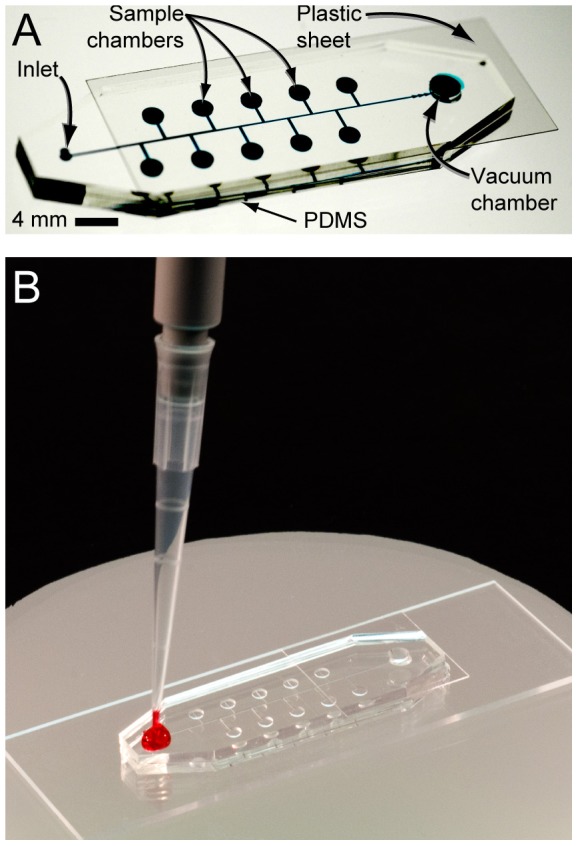
Images of the BacChip. The device consists of 10 microchambers; 9 of the microchambers are preloaded with a different saccharide and one is left empty as a control. We punched holes at the ends of the center PDMS channel to form an inlet port and a vacuum chamber. The PDMS layer was pressed into contact with a glass slide to form the microfluidic system. A plastic sheet was placed over the vacuum chamber to seal it. After removing the device from vacuum it is ready for sample loading by the user. (A) An image of an assembled device in which the channels and chambers are filled with a blue dye to make them visible. (B) After removal from vacuum, the user adds 20 µL of sample to the inlet port and the device fills and isolates cells in the microchambers autonomously.

To perform the bacterial identification assay, a bacterial sample is loaded into a ‘BacChip’ device in one liquid handling step and simultaneously fills multiple microchambers containing different mono-, di-, and trisaccharides (Figure 2). The BacChip is designed to manipulate and isolate the fluid such that each microchamber consists of cells, growth media, and a different concentration of a fermentable saccharide without requiring any moving/mechanical parts or user intervention. After loading, the sample is contained within the device thus insulating the user from potentially harmful organisms. Incubating the BacChip produces visible color changes in the microchambers that are discernable under ambient light. Importantly, the microfluidic platform is flexible and can be fabricated with different numbers and configurations of microchambers with a variety of different assay reagents.

## Materials and Methods

### Bacterial Strains

Strains used in this study–unless otherwise noted–were clinical isolates obtained from stocks cultured in the Department of Medical Microbiology and Immunology (MMI) at the University of Wisconsin–Madison, including: *Enterococcus faecalis* (no strain information available), *Enterococcus faecium* (A634; from Bernard Weisblum, Department of Pharmacology, University of Wisconsin–Madison), *Staphylococcus aureus* (ATCC 25923 and ATCC 13565; also an FRI-100 isolate from the Food Research Institute, Madison, Wisconsin, USA), *Klebsiella pneumoniae* (no strain information available), *Acinetobacter baumannii* (no strain information available), *Pseudomonas aeruginosa* (ATCC 14207), *Enterobacter aerogenes* (ATCC 13048*), Enterobacter cloacae* (an isolate from the Wisconsin State Lab of Hygiene from John Lindquist, Department of Bacteriology, University of Wisconsin–Madison via MMI). Strains were cultured in Luria Bertani (LB) broth at 37°C in a static incubator.

### Fabrication of Devices

BacChips were fabricated by modifying a recently described procedure [4]. In summary, we fabricated masters in SU-8 photoresist (Microchem) on silicon wafers using photolithography. The microchambers were 300-µm tall and 2-mm in diameter with a volume of ∼1 µL (this volume includes the side channel that connects the microchamber to the main channel). The central channel was 50-µm tall, 150-µm wide and 35-mm long. Small triangular features (150–260 µm from tip to base and 50-µm tall) were included at the end of the main channel–closest to the vacuum chamber–to function as a passive one-way valve that prevented the backflow of fluid out of the vacuum chamber after the device was inoculated. We molded a layer of PDMS (Sylgard 184, Dow Corning) with the channels and microchambers using the techniques of soft lithography [16], [17]. A 3-mm diameter hole was punched through the PDMS slab to create the vacuum chamber at one end of the central channel, and a 1-mm diameter hole for the inlet port was punched at the other end of the central channel using tissue biopsy punches (Harris UniCore, Tedpella).

The BacChip consisted of 10 microchambers that contained one of nine different saccharides (cellobiose, glucose, lactose, mannitol, mannose, raffinose, sucrose, trehalose, and xylose) and one control (no saccharide). We chose this particular collection of saccharides because they are differentially fermented by the ESKAPE strains that we assayed in this study [9]–[11]. We delivered a 2-µL aliquot of an aqueous saccharide solution (1% w/v) to each microchamber and dried the solutions at ambient temperature. The PDMS slab containing the microchambers was pressed against a glass slide or cover slip to seal the microchambers and form the BacChip. A plastic cover slip was placed on top of the device to seal the vacuum chamber, leaving only the inlet port uncovered. Fully assembled BacChips were incubated in a vacuum for 30–45 min at ∼200 mTorr prior to loading samples. Once removed from vacuum, an identification assay was initiated by covering the inlet port with a 20-µL drop of a bacterial suspension. This suspension was then drawn into the central microchannel and transported into the microchambers via degas-driven flow [4], [18], [19]. For long-term storage, unused BacChip devices were vacuum-sealed in plastic bags (Foodsaver) using a commercial food sealer (FoodSaver V3835).

### Formulation of the Loading Solution

We formulated a loading solution for transporting cells into microchambers that consisted of a mixture of LB, saline (0.09% NaCl in water, w/v), and 0.05% phenol red (w/v) as a colorimetric indicator of the pH. LB provides critical nutrients that facilitate cell growth and rapid fermentation of the saccharide in the microchamber. We tested loading solutions with LB concentrations (in saline) of 0%, 10%, 25%, 50%, and 100%, added phenol red to a final concentration of 0.05% (w/v), and adjusted the pH to 8.2.

### Optimization of Device Parameters for Identifying Bacteria

We optimized the initial cell concentration and composition of the loading buffer by growing bacteria overnight in liquid LB at 37°C and diluting the culture to an absorbance (λ = 600 nm) that was equivalent to McFarland standards of 0.5 (∼1.5×10^8^ cells/mL), 2.0 (∼6×10^8^ cells/mL), and 4.0 (∼12×10^8^ cells/mL). McFarland standards (McF) are approximations of cell concentration based on the turbidity of the cell suspension. We diluted each of these cultures 100-fold into each of the different loading solution formulations previously described (e.g., 0%, 10%, 25%, 50%, and 100% LB in saline). We also prepared a 1∶200 dilution using the 0.5 McF suspension to test a low concentration of cells (0.75×10^6^ cells/mL), and a single colony that was directly resuspended in loading solution (100 µL) to test a high cell concentration (∼1×10^7^ cells/mL) that can be rapidly formulated.

We loaded 20 µL of each cell dilution into a device and incubated at ambient temperature for ∼15–30 min until the microchambers were filled and isolated, then placed it in a 37°C static incubator. To minimize evaporation within the device during incubation, we placed BacChips in humidity chambers consisting of Petri dishes (150×15 mm, BD Falcon) containing wet paper towels. We observed the devices approximately every hour and recorded the time to identification as the point at which the colorimetric profile of the microchambers reached an initial steady state. This steady state was characteristic of all LB concentrations that we tested and made it possible for us to identify bacteria without continuous colorimetric changes complicating the readout. *A. baumannii* was chosen for the optimization studies with the panel of saccharides we used in this BacChip. Every species of bacteria that we grew using the experimentally optimized conditions gave the same response observed with *A. baumannii*.

### ESKAPE Identification

To identify ESKAPE species, we grew bacteria overnight at 37°C on LB agar, isolated and picked single colonies, and transferred the cells to 100 µL of 25% LB loading solution. We mixed inocula by vortexing, then loaded 20 µL of the cell suspension into a BacChip and incubated at 25°C for ∼15–30 min until the microchambers were filled and isolated. The loaded BacChips were placed in a humidity chamber within a 37°C static incubator and the colorimetric profiles were recorded every 30 min for 6 h. Each species was tested in triplicate. We performed off-chip testing in 1 mL test tubes using identical incubation conditions to confirm BacChip results and the expected outcomes of saccharide fermentation [9]–[11].

## Results

### Optimizing Buffer and Cell Concentration in the BacChip for Bacterial Identification

**Figure 3:**
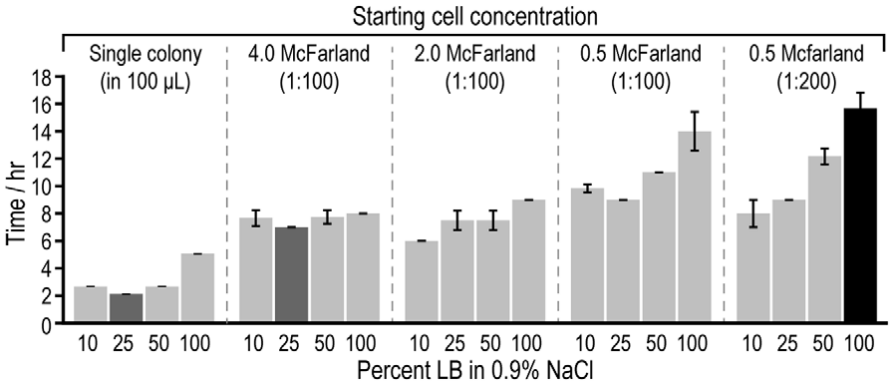
Optimization of buffer conditions and cell concentrations on *A. baumannii* identification. The plot depicts the average time to *A. baumannii* identification using different starting concentrations of cells and loading the BacChip with media formulations consisting of LB diluted in saline. We ran assays with starting cell concentrations of 4.0 McF, 2.0 McF, and 0.5 McF. Parentheses indicate the consequent dilution into the loading media. We also tested single colonies from LB agar plates grown overnight at 37°C that were suspended in 100 µL of loading solution as a starting cell concentration. Based on our sampling time points, our temporal resolution for detection was ∼1 h. The colored bars indicate the most rapid (dark gray) and slowest conditions (black). Error bars indicate the standard deviation of the mean (n≥3).

To benchmark the BacChip against commercial devices, we first optimized the parameters of the assay for the device. We adjusted loading concentrations of cells and LB to increase the rate of acidification of the media within the microchambers and decrease the time to identification. Phenol red is a universal pH indicator that has a bright red (magenta) color above pH 8.6 and is bright yellow below pH 6.8. We found that this indicator transitions from red at pH 8.2 to orange as the pH approaches 6.8. To facilitate the optimization, we scored yellow microchambers as indicative that the cells had metabolized the saccharide to organic acids. In contrast, we scored red or orange microchambers as indicative or no or low saccharide metabolism. While LB provides the nutrients to facilitate rapid growth, it also buffers the loading solution and increases the amount of time before the change in pH can be visualized using phenol red. Furthermore, the production of ammonia from the metabolism of peptone in the media counteracts the acidification that arises from fermentation of the saccharide [20]. We tested solutions of 10%, 25%, 50%, and 100% LB in saline in combination with different cell loading concentrations of *A. baumannii* and determined their effect on the rate of the development of the unique pattern of colored microchambers that was characteristic for this organism. As we typically imaged the pattern formation at 60 min intervals, our time resolution for the development of the pattern was ∼1 h (Figure 3). We also tested saline (0% LB) under different cell loading conditions, but observed that the formation of identification profiles was not reproducible and was accompanied by acidification and a color change in the control chamber due to the formation of carbonic acid in the absence of buffer.

As expected, the most rapid time to identification occurred at low concentrations of LB, with 10% and 25% LB producing the best results. The formulation of loading buffer containing 10% LB produced a mean identification time (n ≥3) of 2.5, 7.5, 6, 9.8, and 8 h at cell concentrations of ∼1×10^7^ cells/mL (colony suspended in 100 µL solution), ∼12×10^6^ cells/mL (100× dilution of 4.0 McF), ∼6×10^6^ cells/mL (100× dilution of 2.0 McF), ∼1.5×10^6^ cells/mL (100× dilution of 0.5 McF), and ∼0.75×10^6^ cells/mL (200× dilution of 0.5 McF), respectively. Loading buffer containing 25% LB produced a mean identification time (n ≥3) of 2, 7, 8, 9, and 9 h at cell concentrations of ∼1×10^7^ cells/mL (colony suspended in 100 µL solution), ∼12×10^6^ cells/mL (100× dilution of 4.0 McF), ∼6×10^6^ cells/mL (100× dilution of 2.0 McF), ∼1.5×10^6^ cells/mL (100× dilution of 0.5 McF), and ∼0.75×10^6^ cells/mL (200× dilution of 0.5 McF), respectively. Even at very low cell concentrations, such as ∼0.75×10^6^ cells/mL, we were able to identify strains within 9 h. The time to identification for lower cell densities was more sensitive to the concentration of LB than higher cell densities. As expected, loading higher cell concentrations into the microchambers reduced the time to identification by increasing the rate of saccharide fermentation. Therefore, we used a high cell concentration–a single colony suspended in 100 µL of loading solution (∼10^6^–10^7^ cells/mL)–for further characterization. The reduced amount of time for the preparation of these samples was advantageous. The 25% LB formulation led to the most rapid identification times over a broad range of starting cell concentrations and was chosen as the loading solution for our identification tests.

### Detection of ESKAPE Pathogens using the BacChip

**Figure 4:**
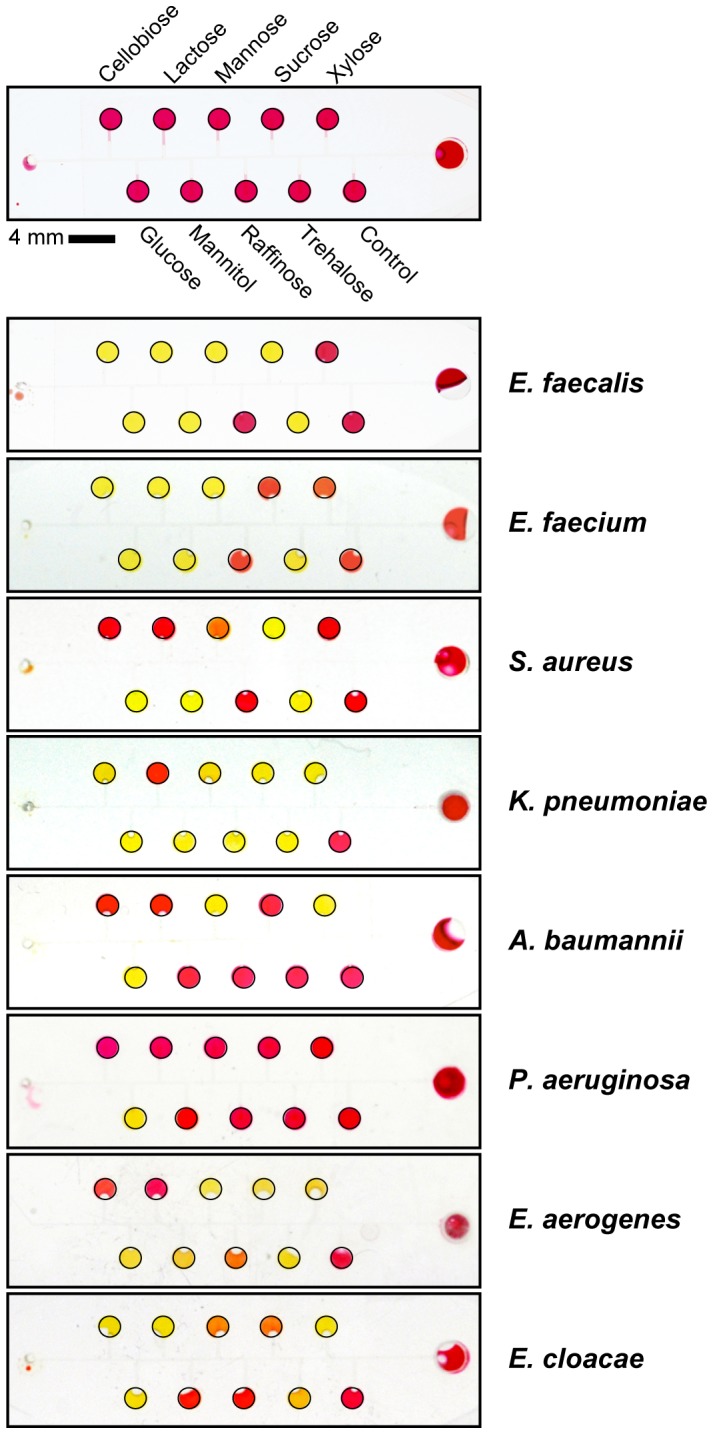
Different colorimetric profiles of ESKAPE pathogens in BacChips. We loaded devices using a single isolated colony suspended in 100 µL of loading media consisting of 25% LB and 0.05% phenol red in saline. We incubated BacChips at 37°C and imaged the devices after 4 h. Yellow microchambers indicate a decrease in the pH below 6.8, which arises from fermentation of the saccharide; red microchambers match the control and indicate no fermentation (pH 8.2). Orange microchambers indicate that the bacterial are slowly metabolizing the saccharide and the pH is >6.8. Black lines were added to the edge of each device and microchamber to aid in visualization.

As indicated in Figure 4, the colorimetric readout of devices that contained a series of nine fermentable saccharides was sufficient to identify individual bacterial strains from a panel of seven ESKAPE strains, and *Enterococcus faecalis*. We inoculated the devices using the optimized conditions described above in which a single colony was suspended in 100 µL of a solution consisting of 25% LB and 0.05% phenol red loading buffer. The colorimetric readout was recorded after an incubation period of 4 h at 37°C for every device, although most strains could be identified in as little as 2 h. Each ESKAPE microorganism produced a unique metabolic fingerprint that was discernable using visible light. We tested each ESKAPE strain in three parallel BacChip devices and found identical results in <4 h. We also performed test tube-based assays on these strains (data not shown) and confirmed reported results of saccharide metabolism [9]–[11]. To test the reproducibility of the colorimetric profiles over a large number of parallel devices, we loaded 30 devices with *A. baumannii* and observed that the metabolic patterns were identical across all devices.

**Figure 5:**
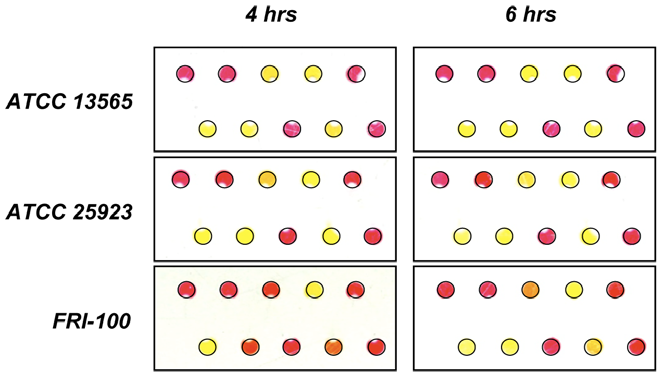
Strain-to-strain variation of *S. aureus* in BacChips. We tested *S. aureus* ATCC 13565, ATCC 25923 and FRI-100 in triplicate. Devices were loaded using a single isolated colony suspended in 100 µL of loading media consisting of 25% LB and 0.05% phenol red in saline. We incubated BacChips at 37°C and imaged the devices after 4 h and 6 h. ATCC 13565 and 25923 developed into a stationary colorimetric profile within 4 h, however FRI-100 required 6 h of incubation. Black lines were added to the edge of each device and microchamber to aid in visualization. The configuration of saccharides and microchambers is as shown in Figure 4.

In addition to the seven ESKAPE pathogens, we tested *Enterococcus faecalis* as it is closely related to *E. faecium* and is clinically important due to its ability to become vancomycin resistant [21], [22]. As expected, they provided similar profiles, however *E. faecalis* was discernable by its ability to ferment sucrose. We also tested *S. aureus* ATCC 25923, ATCC 13565, and FRI-100 for variations in strains of same species. The results demonstrated variability in detecting different species in BacChip assays, and not between strains of bacteria, which is important for reducing the misidentification of species of pathogenic bacteria. When tested in the device, all three strains produced an identical colorimetric profile that is characteristic of *S. aureus*. Both ATCC 25923 and ATCC 13565 were identifiable within 4 h, whereas FRI-100 was only identifiable at 6 h as it fermented mannose, mannitol, and trehalose slower than the other two strains (Figure 5). Although experimentally untested, we believe that increasing the loading concentration of cells (e.g., one colony suspended in 50 µL of loading solution) can reduce the time to identification to within 4 h. Colony fitness and cell density may also have played a role in the time required to identify strain FRI-100.

## Discussion

This report describes a portable, self-loading microfluidic device that can distinguish between ESKAPE pathogens in <4 h. Importantly, the projected cost of BacChips is low (<$1.50 based on purchasing the materials at retail value) because they are small, plastic, and can be fabricated by embossing and other techniques that are compatible with rapid prototyping; costs can be dramatically reduced by purchasing materials in high volume and using large volume manufacturing. Vacuum-sealed BacChips remain functional for at least two weeks; experiments are currently underway to test the lifetime of vacuum-sealed devices and to optimize this step using thicker plastic bags and a larger source of negative pressure. These devices can be conveniently stored at room temperature, as the rate of saccharide degradation is slow in the solid state under these conditions. Strains were identifiable even at low cell concentrations (∼0.75×10^6^ cells/mL). We did not determine the limit of detection in the BacChip, as clinical microbiological assays require colony isolation and amplification of bacteria from samples prior to testing, and hence cell density is typically not limiting.

Importantly, BacChips require only one pipetting step to load and operate the device. The device is designed such that the transport and isolation of a sample in the microfluidic system occurs without the mixing of dissolved reagents in each microchamber. Additionally, having observed consistent results by loading a single colony suspended in buffer, we were able to eliminate extra steps and equipment needed to standardize the cell concentration in the loading mixture. While the basic microfluidic technology has not changed from our previous work [4], we have demonstrated the flexibility of this platform as a stepping-stone to future applications in clinical microbiology. In principle the microchambers of BacChips can be loaded with any reagent, such as antibiotics for measurements of susceptibility and minimum inhibitory concentration [4], as well as biomolecules and synthetic small molecules for applications in biosensing, biotechnology, and drug discovery. The high surface area-to-volume ratio of the microchambers and the permeability of PDMS to gases also support fast growth rates, making this an excellent platform for other biological assays [23].

Some areas of the BacChip can be further improved. For example, increasing the number of microchambers containing carbon sources may provide additional variability between different organisms and strains and increase the database correlating metabolic fingerprints to different genotypes. We used BacChips to profile ESKAPE pathogens, however, the device does not currently have the capacity to identify thousands of unique organisms, as is possible with other commercial devices. Yet, the devices are easily expandable and can be designed to include hundreds of microchambers that test for additional saccharides and metabolites, increasing the specificity of each strain tested and potentially contributing to a much larger profile database than is currently available commercially. We have designed and implemented a BacChip containing 30 microchambers that can either be used to assay bacterial growth against 29 saccharides or simultaneously test a sample against a panel of 9 saccharides in triplicate. The devices are also easily customizable and can be preloaded with a variety of dried reagents to perform additional assays in parallel with saccharide-based identification. For example, devices can be designed to simultaneously identify strains and measure antibiotic susceptibility by including microchambers that contain varying concentrations of antibiotics to test for minimum inhibitory concentrations [4].

We observed an initial steady state for the color of the microchambers at ∼4 h that provided a convenient time point for the assay readout. We assume that this lag is the result of diauxic growth and represents a transition between primary and secondary metabolism. Not surprisingly, additional incubation led to a ‘second stage’ arising from secondary metabolism that decreased the pH in many of the microchambers and turned the indicator yellow. The lag time between the first and second stage was dependent on the LB composition of the loading solution and varied from 2–10 h. Due to its buffering capacity, decreasing the concentration of LB increased the number of microchambers that became acidic during the second stage. In contrast, devices loaded with 100% LB showed the opposite effect. This observation may be explained by the production of ammonia during metabolism of peptone, which buffers the pH of the liquid in microchambers during later stages of growth.

Strain-to-strain variability was a concern for the BacChip assay, as it is possible that different strains of the same species will produce different profiles within the device. As the current configuration of this BacChip has only 9 identifiers, uncommon strains within the same species may be misidentified. To explore the strain-to-strain variability between devices, we tested three different strains of *S. aureus* (ATCC 25923, ATCC 13565, and FRI-100) and observed the same colorimetric profile for all three strains (Figure 5).

Reducing the time from the start of the assay to identification is important for diagnostic assays. In particular, reducing the delay for the detection of a pathogen can improve patient outcome and reduce the costs associated with the treatment of infections [4], . The BacChip enables the rapid identification of bacteria, which complements commercially available technologies for identification and susceptibility testing. One approach to reduce the time for the identification of bacteria using the BacChip is to replace phenol red with a different colorimetric indicator that develops rapidly. For example, we observed that incorporating bromothymol blue into the optimized loading parameters reduced the identification by as much as one hour in several of our BacChip ESKAPE assays. Alternatively, the use of a fluorescent indicator of metabolism (e.g., a tetrazolium salt) enables fluorescence detection, which is typically an order of magnitude more sensitive than absorbance, and could thus reduce the time associated with the detection step. In principle BacChips can be engineered to fit inside a fluorescence plate reader and the spacing of the microchambers can be designed to match the well footprint of 96, 384, or 1536 well plates.

In addition to reducing the time to identification, the advantages of these devices include their portability, limited reagent consumption, and the lack of external instrumentation required for their operation. The current size of this BacChip makes it small enough to fit inside a shirt pocket; reconfiguring the number, dimensions, and layout of microchambers could further reduce the footprint of the devices. Furthermore, it should be possible to multiplex assays, such as simultaneously detecting bacteria in a sample and measuring their susceptibility to antibiotics. The ESKAPE panel of pathogens represents an important group of bacteria that are relevant to clinical infections, including organisms that have developed resistance to many classes of therapeutic antibiotics. As a portable diagnostic device, the BacChip may serve as a useful tool in point-of-care and distributed medicine, where it may guide the effective prescription of antibiotics.
